# *Hygrophorus eburneus*, edible mushroom, a promising natural bioactive agent

**DOI:** 10.17179/excli2019-2056

**Published:** 2020-03-31

**Authors:** Marijana M. Kosanic, Dragana S. Šeklic, Milena M. Jovanovic, Nevena N. Petrovic, Snežana D. Markovic

**Affiliations:** 1 Faculty of Science, Department of Biology and Ecology, University of Kragujevac, Radoja Domanovica 12, 34000 Kragujevac, Serbia

**Keywords:** anticancer, antimicrobial, antioxidant, edible mushroom, neuroprotective

## Abstract

It is known that many edible mushrooms have important medicinal properties, including effects on different types of cancers. This is the first report regarding the neuroprotective, antimicrobial, antioxidative and anticancer activities of the acetone extract of edible mushroom *Hygrophorus eburneus. *Neuroprotective potential was evaluated by measuring the capacity of the extract to inhibit acetylcholinesterase. In this assay, the tested extract showed activity against acetylcholinesterase in a dose-dependent manner where the percentage of inhibition ranged from 13.19 to 46.44 %. The antimicrobial potential was determined by the microdilution method against five species of bacteria and eight species of fungi and the results of this method exhibited moderate antimicrobial activity of *H. eburneus *with MIC values ranging from 6.25 to 25 mg/mL. Antioxidant activity was evaluated by measuring the scavenging capacity of the tested sample on DPPH and superoxide anion radicals, by the reducing power assay and by measuring the amounts of total phenolics in extract. As a result of the study,* H. eburneus *extract showed a potent antioxidant activity (IC_50 _were 102.93 μg/mL for DPPH radical scavenging activity and 123.27 μg/mL for superoxide anion radicals scavenging) while absorbances for reducing power assay were from 0.0235 to 0.1161. The total phenolic content in the extract was 9.27 µg PE/mg. Finally, anticancer effects were evaluated by MTT test for cytotoxicity, acridine orange/ethidium bromide staining for detection of the type of cell death and wound healing assay for antimigratory effects on human colorectal cancer cell line (HCT-116) and human breast cancer cell line (MDA-MB-231). The results for cytotoxicity and apoptosis were measured after 24 and 72 h and for anti-migratory effect after 12 and 24 h. The tested *H. eburneus *mushroom extract expressed cell selectivity, with notable cytotoxic effects observed on HCT-116 cells, with a strong proapoptotic potential. The migration of HCT-116 cells was significantly inhibited, while MDA-MB-231 cells were less sensitive to the treatment. The results of this study revealed that the tested extract had relatively strong neuroprotective, antimicrobial, antioxidant, and anticancer effects. It suggests that this mushroom can be proposed as a novel source of nutraceuticals and pharmaceuticals.

## Introduction

From the ancient time, wild edible mushrooms have been an integral part of human diet worldwide. Today, mushrooms are valuable, not only because of their unique flavor and texture, but because of their important nutritional characteristics. Nutritional value of edible mushrooms is highly significant, since they are rich in proteins (including all the essential amino acids), dietary fiber, essential oils, minerals, and vitamins. In addition to that, they are low in calories, fat and cholesterol (Ouzouni et al., 2009[31]; Boonsong et al., 2016[6]; Friedman, 2016[10]). Apart from their nutritional value, many edible mushrooms can be used in therapeutic purposes. Bioactive compounds in edible mushrooms, often called nutraceuticals, are the reason why so many edible mushrooms can be considered functional food and can be used in prevention and even treatment of many diseases. Lectins, polysaccharides, triterpenoids, phenolics, flavonoids, tocopherols, ascorbic acid, carotenoids, ergothioneine, glutathione, and selenium are just some of the nutraceuticals that can be found in some species of edible mushrooms (Lakhanpal and Rana, 2005[24]; Kalaras et al., 2017[15]). 

Numerous studies have showed that many edible mushrooms have important medicinal properties, such as: anticancer, immunomodulatory, anti-inflammatory, antidiabetic, antiviral, antioxidant, antimicrobial, antineurodegenerative, antihypertensive, cholesterol-lowering, etc. (Ajith and Janardhanan, 2007[1]; Pandimeena et al., 2015[34]; Kosanić et al., 2016[21]; Özdal et al., 2019[33]). They have been recently characterized as effective natural remedies for colorectal carcinoma (CRC), with effects on different mechanisms in cancer cell lines (Šeklić et al., 2016[39]). Therefore, cancer fungotherapy, as a promising scientific field aimed at more specific anticancer activity of mushrooms, is beginning to intensify (Blagodatski et al., 2018[5]). Many literature sources (Kim et al., 2013[18]; Wang et al., 2014[45]; Li et al., 2014[25]; Šeklić et al., 2016[39]) confirmеd the ability of different fungal species to prevent cancer genesis, their tumor-suppressing activity and growth inhibitory effects (especially on gastrointestinal cancers), with anti-metastatic activity.

In this study we inspected bioactive properties of *Hygrophorus eburneus*, the ivory waxcap, an edible, but not so popular species of mushroom. Fruiting bodies of this species are medium-sized, pure white and often covered with a thick layer of slime, during moist conditions, usually growing solitary or subgregarious in hardwood and conifer forests, commonly during autumn months. 

Considering that* H. eburneus* is an edible mushroom species, which occurs in abundance, it is important to recognize its bioactive properties. However, the data about its bioactivity is limited and very scarce. Only one report analyses *H. eburneus* and describes the antibacterial and antifungal properties of eight bioactive fatty acids isolated from this species (Teichert et al., 2005[41]). Since there аre many well-known health benefits of mushrooms, but there are no available data on the bioactivity of *H. eburneus*, we designed this study to gain insight into the bioactive potentials of this species. Therefore, in this research we examined the neuroprotective, antimicrobial, antioxidative, and anticancer potentials of *H. eburneus* acetone extract (HEAE) in order to discover if this mushroom can be used as functional food and a possible natural drug for the treatment of various diseases.

## Materials and Methods

### Collection of fungal samples and preparation of the extract 

Samples of fruiting bodies of *Hygrophorus eburneus* (Bull.) Fr., were collected in Niš, Serbia, in 2017. Voucher specimens, labeled DBFS95 are deposited and stored in the facility of the Department of Biology and Ecology, Faculty of Science, Kragujevac, Serbia. The identification of the collected samples was done using standard literature (Uzelac, 2009[44]).

Finely dried and powdered mushroom samples were extracted using acetone as a solvent, in a Soxhlet extractor. After that, the extract was filtered and concentrated under reduced pressure in the rotary evaporator (IKA, RV 10, Werke, Staufen, Germany). Before its application in the experiments, the dry extract was stored at -18 °C. For cell analyses, HEAE was dissolved in DMSO (dimethyl sulphoxide) and DMEM (Dulbecco's Modified Eagle Medium) (1:10) to obtain stock solution (1 mg/mL). Applied concentrations, where final DMSO was less than 0.05 % without cytotoxic effects on cells (Hostanska et al., 2007[13]), were generated by further dilution in DMEM. For other *in vitro *experiments, the extract was dissolved in 5 % DMSO. The desired concentration of DMSO was adjusted by adding sterile distilled water. 

### The evaluation of acetylcholinesterase inhibition 

The effect of HEAE on the acetylcholinesterase (AChE) inhibition rate was measured spectrophotometrically, using 96-well microtiter plates, according to the method of Ellman et al. (1961[8]). Acetylcholine iodide (AChI) was used as the substrate for the enzyme AChE, which degrades this compound to acetate and thiocholine. In the next step, 5,5′-dithiobis (2-nitrobenzoic acid) (DTNB) was transformed with thiocholine to the yellow-colored 5-thio-2-nitrobenzoate anion (TNB^2-^) and the change in absorbance was recorded on ELISA microplate reader (RT-2100C, Hamburg, Germany), at a wavelength of 412 nm and temperature of 25°C. The measured values were compared to galanthamine, the commercial inhibitor of AChE. 

### Antimicrobial activity

The antimicrobial activity of HEAE was tested on five species of bacteria: *Staphilococcus aureus *(ATCC 25923),* Bacillus subtilis *(ATCC 6633),* B. cereus *(ATCC 10987),* Escherichia coli *(ATCC 25922) and* Proteus mirabilis *(ATCC 12453); and eight species of fungi: *Aspergillus flavus *(ATCC 9170),* A. fumigatus *(ATCC 1022),* Candida albicans *(ATCC 10259),* Geotrichum candidum *(ATCC 34614),* Trichophyton mentagrophytes *(ATCC 9533),* Fusarium solani *(ATCC 36031), *Penicillium chrysogenum *(ATCC 18502) and* Paecilomyces variotii *(ATCC 10106). All cultures were provided from the American Type Culture Collection (ATCC). Bacterial isolates were picked from over-night cultures in Müller-Hinton agar and the suspensions were prepared with sterile distilled water, by adjusting the turbidity to match 0.5 McFarland standards which is approximately 10^8^ CFU/mL. Fungal suspensions were made from 3- to 7-day-old cultures maintained on potato dextrose agar, except *C. albicans*, which was maintained on Sabouard dextrose (SD) agar. The spores were rinsed with sterile distilled water, used to determine turbidity spectrophotometrically at 530 nm, and then further diluted to approximately 10^6 ^CFU in accordance with the procedure approved by NCCLS (1998[28]). Minimum inhibitory concentrations (MIC) were evaluated by the 96-well microtiter assay using resazurin as the indicator of cell growth (Sarker et al., 2007[38]). Standard antibiotics (streptomycin for bacteria and ketoconazole for fungi) were used as positive controls, while DMSO was used as a negative control. 

### Antioxidative activity

Antioxidant activity of HEAE was evaluated applying free radical scavenging, superoxide anion radical scavenging and reducing power assays. The free radical scavenging activity was measured by 1,1-diphenyl-2-picryl-hydrazil (DPPH) method, according to Kosanić et al. (2016[21]). The superoxide anion radical scavenging activity was studied according to the method of Nishikimi et al. (1972[29]). The Oyaizu method (1986[32]) was used to determine the reducing power of the extract. In all antioxidant assays, ascorbic acid was used as a positive control. In addition, the estimation of total phenolic content in HEAE was done in accordance with the method of Slinkard and Singleton (1977[40]), using pyrocatechol as the standard phenolic compound. 

### Methodology of cell cultivation and anticancer analysis

The colorectal carcinoma HCT-116 and breast carcinoma MDA-MB-231 cell lines were obtained from the American Tissue Culture Collection (Manassas, VA, USA). Cells were maintained in DMEM, supplemented with 10 % fetal bovine serum (Gibco, USA) and antibiotics (100 IU/mL penicillin and 100 μg/mL streptomycin) (Invitrogen, USA). Cells were grown in standard culturing conditions and after a few passages were seeded in appropriate plates for different assays. 

HCT-116 and MDA-MB-231 cells were treated with the extract in concentration range of 1, 10, 50, 100, 250 and 500 μg/mL for MTT assay. Concentrations of 100 and 250 μg/mL were used for determination of the type of cell death (AO/EB assay). Results were measured after 24 and 72 h. Concentrations used for wound healing assay were 10 and 100 μg/mL and the results were measured after 12 and 24 h of treatments.

### Cell viability assay (MTT assay) 

Cell viability was determined by MTT assay (Mosmann, 1983), according to the procedure previously described by Šeklić et al. (2016[39]). This assay is based on the color reaction of mitochondrial dehydrogenase of the living cells with 3-[4,5-dimethylthiazol-2-yl]-2,5-diphenyltetrazolium bromide (MTT, Sigma, USA). The evaluation of cytotoxic effects of HEAE was based on the obtained IC_50 _values, which represent the treatment doses inducing the death of 50 % of treated cells.

### Fluorescence microscope analysis of cell death (AO/EB) assay 

In order to determine the type of cell death, Acridine orange/ethidium bromide (AO/EB) double staining assay was used (Baskić et al., 2006[4]), according to the detailed protocol already described by Kosanić et al. (2014[22]). Acridine orange is a fluorescent dye taken up by both viable and nonviable cells emitting green fluorescence when intercalated into double stranded nucleic acid (DNA), or red fluorescence when the dye bounds to single stranded nucleic acid (RNA). Meanwhile, ethidium bromide is taken up only by nonviable cells and fluoresces red, after bonding with DNA. According to the occurrence of fluorescence and the morphological aspect of chromatin condensation in the stained nuclei, it is possible to distinguish four types of cells: viable cells with uniform bright green nuclei and organized structure; early apoptotic cells with green nuclei, with chromatin condensation visible as bright green patches or fragments; late apoptotic cells with orange to red nuclei and condensed or fragmented chromatin.; necrotic cells with uniformly orange to red nuclei and condensed structure. To each well, 20 μl of dye mixture (10 μl/mg AO and 10 μl/mg EB in distilled water) was added and micrographs were taken using Nikon inverted fluorescent microscope (Eclipse Ti) (Nikon Instruments Inc., Melville, NY, USA) at 400× magnification*. *In every sample a minimum of 300 cells was counted.

### Wound healing assay (Scratch assay) 

Cell migration was evaluated *in vitro* using a method based on making a scratch in a confluent cell layer and monitoring the behavior and movement of cells over time (He et al., 2016[11]). Cells located at the edge of the wound will begin to move in order to fill in and close the "wound". The degree of cell migration was determined by comparing the images created at the moment of making the wound (0 h) and the ones created at the end of a certain period (12, 24 h). First of all, HCT-116 and MDA-MB-231 cells were seeded in a 12-well plate (7 x 10^5 ^cells per well) and after 24 h, when cells reached 90-100 % confluence, the growth medium was aspirated and a scratch in the confluent cell monolayer was made using a plastic disposable pipette tip (100 µL). Cells were rinsed twice with PBS to remove detached cells and then treated with 1 mL of investigated HEAE in the appropriate concentration (10 and 100 µg/mL). Micrographs were taken using inverted NICON Eclipse Ti microscope at 100x magnification. The distance between cells, located at the edges of the wound, was measured using ImageJ software package and calculated as a ratio of the values of the treated group divided by the values of the control group, multiplied by 100 in order to obtain the percentage of the relative wound area.

### Statistical analysis 

Statistical analyses were performed using Microsoft Excel and SPSS software packages. The data for cell analysis are expressed as means ± standard error (mean ± SE) of three independent experiments, while the data for *in vitro* experiments are expressed as means ± standard deviations (mean ± SD) of three parallel measurements. IC_50 _values were calculated by a computer program CalcuSyn. Student's *t*-test was used in determining the statistical significance of the activity of the tested extract.

## Results

**Figure 1:**
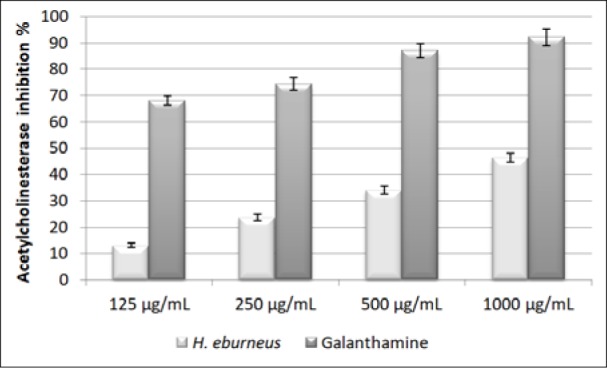
Acetylcholinesterase inhibition of HEAE. Values are expressed as mean ± SE of three parallel measurements. There is statistically significant difference between tested mushroom extract and control (p<0.05).

The obtained results of the evaluation of acetylcholinesterase inhibition are shown in Figure 1(Fig. 1). Compared to galanthamine, HEAE showed moderate effect on the inhibition of AChE*.* The percentages of obtained values of the inhibition of AChE by HEAE were 46.44, 34.06, 23.66 and 13.19 %. As shown, the rate of inhibition of AChE depended on the concentration of the mushroom extract.

The antimicrobial effect of the mushroom extract on the tested microorganisms is shown in Table 1(Tab. 1). The HEAE affected all tested microorganisms. The MIC values varied from 6.25 to 12.5 mg/mL for bacteria and from 6.25 to 25 mg/mL for fungi. The most susceptible microorganisms were *B. subtilis, B. cereus, E. coli, P. mirabilis* and *A. fumigatus* (MIC values were 6.25 mg/mL), while the most resistant was *P. chrysogenum *with MIC value of 25 mg/mL. The antimicrobial activity of the mushroom was compared to standard antibiotics, streptomycin (for bacteria) and ketoconazole (for fungi). The results showed that standard antibiotics were more active than the tested mushroom. DMSO was used as the negative control and it showed no effect on the tested microorganisms.

The results of DPPH radical scavenging, superoxide anion radical scavenging activity, and reducing power assays of the studied extract are presented in Table 2(Tab. 2). The IC_50_ values were 102.93 μg/mL for DPPH assay and 123.27 μg/mL for superoxide anion radical scavenging activity assay. As shown in Table 2(Tab. 2), the reducing power was concentration-dependent. Absorbance values in reducing power assay varied from 0.0235 to 0.1161. In addition, the total phenolic content in the extract was 9.27 µg PE/mg (Table 2(Tab. 2)). The analyses of these various antioxidant activities revealed that there was a statistically significant difference between the extract and the control (P < 0.05).

**Figure 2:**
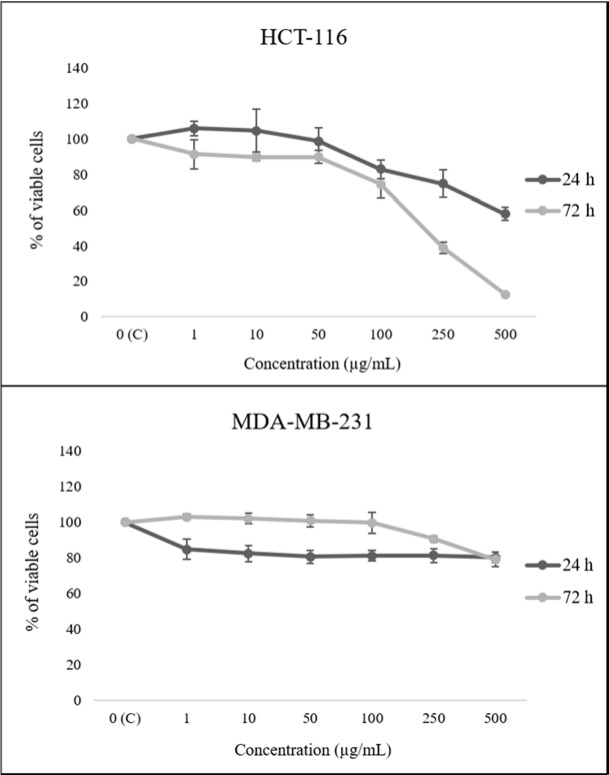
The effect of HEAE on viability of HCT-116 and MDA-MB-231 cells. Results are presented as means ± SE of three independent experiments.

The HEAE reduced HCT-116 cell viability in a dose- and time-dependent manner, expressing a stronger effect after 72 h, while up to 80 % of cell viability of MDA-МB-231 cells was reduced only after 24 h (Figure 2(Fig. 2)). The extract showed the most prominent cytotoxic effect on HCT-116 cells with IC_50_^72h ^value of 178.54 μg/mL (Table 3(Tab. 3)). Meanwhile, the reduction of MDA-МB-231 cells viability was insignificant, which is in correlation with presented IC_50_ values (Table 3(Tab. 3)).

**Figure 3:**
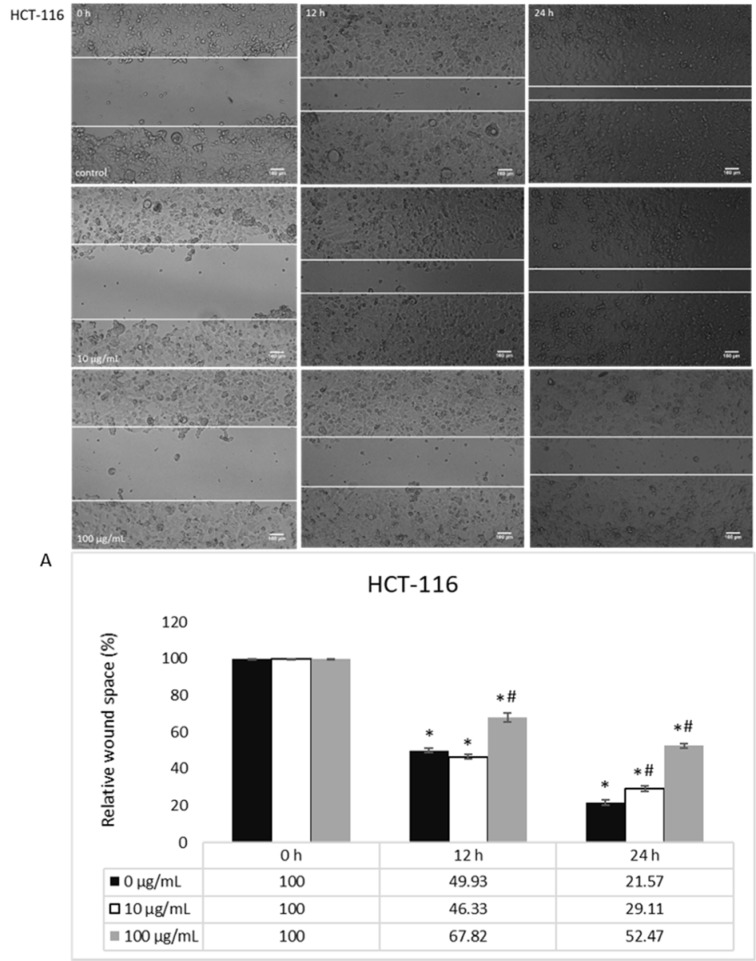
The effect of HEAE on migratory potential of HCT-116 cells; A) Analysis of wound space by ImageJ software and shown as relative level of changes compared to control cells (100 %). Results are presented as means ± SE of two independent experiments. *p<0.05 is considered as statistically significant difference compared to control (0 h) for treatment, and ^#^p<0.05 statistically significant difference between treatments compared to control (0 μg/mL) for the same time.

**Figure 4:**
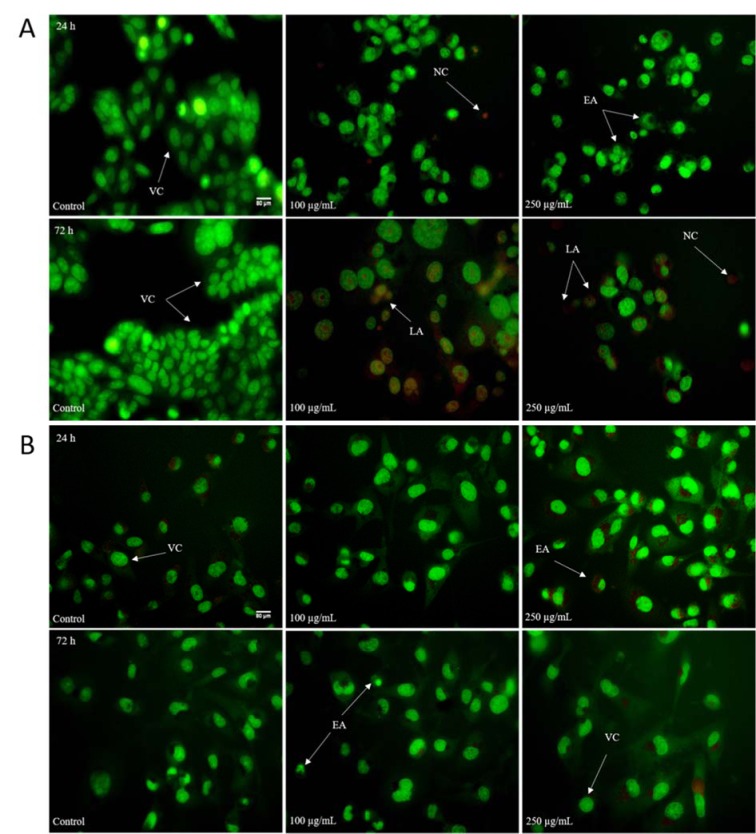
Morphological changes of HCT-116 (A) and MDA-MB-231 (B) cells stained with AO/EB after exposure to the HEAE. VC - viable cells; EA - early apoptosis; LA - late apoptosis; NC - necrotic cells. Untreated cells served as control.

The above presented data showed that HEAE induced cell death mainly by apoptosis (Table 4(Tab. 4), Figure 3(Fig. 3)) followed by typical apoptotic morphological changes (reduction of cell size, blabbing effects with condensation and fragmentation of nuclear material and formation of apoptotic bodies). The treatment was more effective on HCT-116 cells (Figure 4A(Fig. 4)), with the strongest effect after 72 h. The higher dose (250 μg/mL) of HEAE induced significant proapoptotic activity after 24 h, with a total of 50.29 % of apoptotic HCT-116 cells (green colored cells) and 2.12 % of necrotic cells (homogeneous red particles) (Table 4(Tab. 4), Figure 4A(Fig. 4)). After 72 h, the percentage of cells in early apoptosis was 27.86, 23.14 % cells in late apoptosis and 24.51 % in necrosis (Table 4(Tab. 4)). This significant, proapoptotic characteristic of HEAE is in correlation with its cytotoxic potential against HCT-116 cell line (Figure 2(Fig. 2), Table 3(Tab. 3)). When it comes to MDA-MB-231 cell line, an acute proapoptotic effect was detected after 24 h, with a significant percent of early apoptotic cells, but this effect was reduced after 72 h (Table 4(Tab. 4), Figure 4B(Fig. 4)), confirmed by results of cytotoxicity (Figure 2(Fig. 2)).

**Figure 5:**
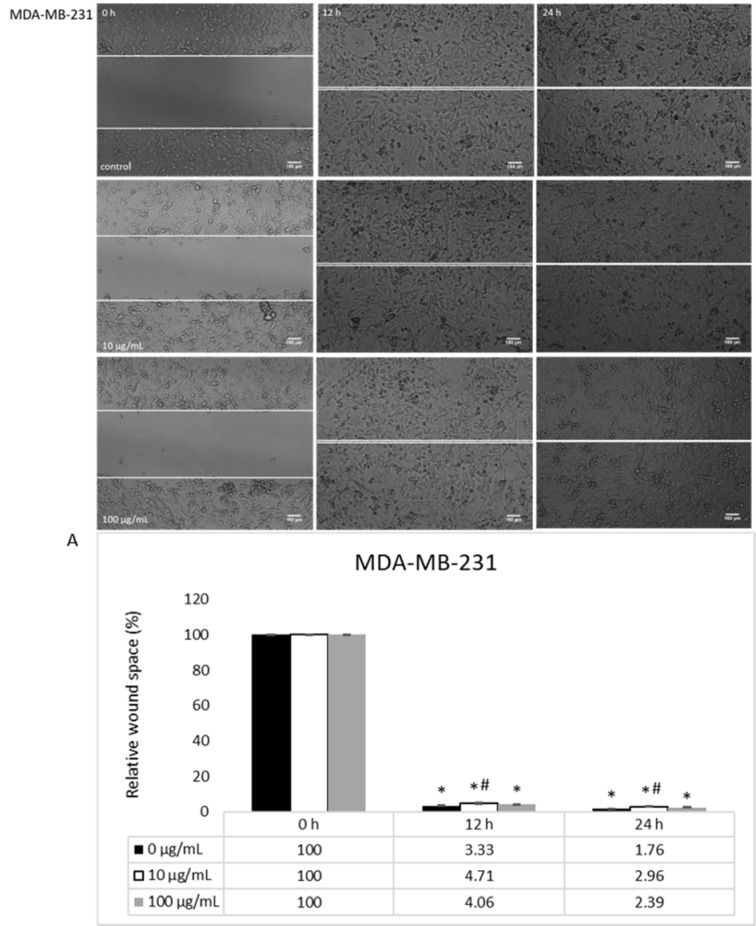
The activity of HEAE on migratory potential of MDA-MB-231 cells; A) Analysis of wound space using ImageJ software and shown as relative level of changes compared to control cells. Results are presented as means ± SE of two independent experiments.*p<0.05 is considered as statistically significant difference compared to control (0 h) for treatment, and ^#^p<0.05 statistically significant difference between treatments compared to control (0 μg/mL) for the same time.

The basal migration of untreated HCT-116 and MDA-MB-231 cells, both with prominent migratory potential, was measured after 0, 12 and 24 h, where MDA-MB-231 cells had a notably enhanced migration (Figures 3(Fig. 3), 5(Fig. 5)). When it comes to the treatment, HEAE was applied in two selected concentrations (10 and 100 μg/mL), which significantly inhibited HCT-116 cells migration in a dose- and time-dependent manner (Figure 3(Fig. 3)). HEAE in concentration of 100 μg/mL inhibited HCT-116 cells migration after both treatment incubation periods for 30 % (Figure 3A(Fig. 3)), while the concentration of 10 μg/mL showed effect only after 24 h. It was shown that higher concentrations strongly inhibited cell migration. Although the basal MDA-MB-231 cell line motility is high (compared to HCT-116 cells), the tested HEAE exerted inhibition of MDA-MB-231 cell motility even in concentration of 10 μg/mL, compared to control (Figure 5(Fig. 5)). 

For further results see the Supplementary data.

## Discussion

### Neuroprotective, antimicrobial, antioxidant, and anticancer potentials of HEAE were presented in this study.

Although the etiology of neurodegenerative disorders, primarily Alzheimer's disease, has not been fully explained, it is known that at the base of these diseases is a decreased level of acetylcholine i.e dopamine. Enzyme AChE hydrolyses the neurotransmitter acetylcholine, thereby stopping the synaptic transmission. Therefore, AChE inhibitors are considered to be the most effective agents in the treatment of these disorders due to the fact that reduction of this enzyme's activity helps restore the level of acetylcholine in cholinergic synapses. Since synthetic inhibitors of AChE are expensive and usually have various side effects, more attention is paid to finding natural, alternative sources. In this study, *H. eburneus* has been tested in terms of inhibition of AChE activity for the first time. Previous studies have reported that extracts of few mushroom species, including seven wild mushroom species, belonging to genus *Polyporus* and three other mushroom species (*Cantharellus cibarius*, *Lactarius deliciosus* and *Trametes versicolor*), contain compounds which inhibit AChE activity (Orhan and Üstün, 2011[30]). In comparison with the AChE activity inhibition rate, which was obtained by studying the above mentioned mushrooms (6.81-37.61 % at concentration of 0.5 mg/mL), HEAE can be considered as a good neuroprotective agent against Alzheimer and other similar diseases. This activity of mushrooms is attributed to terpenoids and alkaloids, which inactivate AChE by binding to the active center or peripheral binding sites (Patocka, 2012[35]). Also, phenolic acids and flavonoid derivatives have been reported to be potent inhibitors of AChE (Roseiro et al., 2012[36]). 

There is a constant need for novel antimicrobial compounds because of the growing resistance of pathogens and development of new diseases. According to the previously published data (Heleno et al., 2015[12]; Kosanić et al., 2016[21]; Rosenberger et al., 2018[37]; Özdal et al., 2019[33]), mushrooms can exhibit antimicrobial effect on a great number of microorganisms. Therefore, the present study represents HEAE as a possible source of bioactive compounds possessing an antibiotic activity. Prior to our research, eight bioactive fatty acids were isolated from *H. eburneus* and it was proven that they possessed antibacterial and antifungal properties (Teichert et al., 2005[41]). 

Apart from that, there are no other data on antimicrobial activity of this mushroom. As a result of our study, HEAE can be claimed to have an impact on all tested microorganisms at moderate doses. The intensity of the antimicrobial effect depended on the species of the tested microorganisms and the used concentration of HEAE. In this research, the most sensitive bacterial strains were *B. subtilis, B. cereus, E. coli *and* P. mirabilis* while* A. fumigatus* was the most sensitive fungal strain. The probable mechanisms of antimicrobial activity of the tested extract are the inhibition of cell wall synthesis, the inhibition of protein synthesis, the alteration of cell membranes and the inhibition of nucleic acid synthesis. In all cases, the cells become unable to function normally (Bajpai, 2016[3]). In this experiment, antimicrobial effect was observed against both bacteria and fungi, with fungi being the more resistant one. Fungi were assumed to be more resistant to the tested extract than bacteria due to more complex structure of the cell wall. This observation is in accordance with many other studies focused on antimicrobial activity (Heleno et al., 2015[12]; Kosanić et al., 2016[21]; Rosenberger et al., 2018[37]), which have demonstrated that the structure and the permeability of the cell wall are main reasons for different sensitivities in bacteria and fungi.

Antioxidants have a vital role in the elimination of reactive oxygen species, whose increased levels lead to oxidative stress and disorders of the oxidative homeostasis. Various synthetic antioxidants were discovered to be toxic and even carcinogenic. Therefore, there is an expanding need for finding natural alternatives (Naveena et al., 2008[27]; Kosanic and Rankovic, 2015[19]). Antioxidant activities of many species of mushrooms have been proven throughout several studies (Boonsong et al., 2016[6]; Kosanic et al., 2016[21]; Özdal et al., 2019[33]), leading to a conclusion that mushrooms could serve as an alternative source of antioxidants. Antioxidative properties of mushrooms are related to their bioactive compounds such as: phenolics, flavonoids, glycosides, polysaccharides, tocopherols, ergothioneine, carotenoids, and ascorbic acid (Kozarski et al., 2015[23]). Various studies have shown that phenolics are the major antioxidants in mushrooms (Ferreira et al., 2007[9]; Kosanić et al., 2017[20]). In accordance to that, our results show that the content of phenolics in HEAE is relatively high, which means that these compounds are probably responsible for the detected antioxidative activities. On the other hand, it is difficult to compare our results to the results of other authors, due to differences in the applied extraction method, the use of different extraction solvents, the mode of demonstrating the results (on dry or fresh basis of mushrooms), the evaluation method, etc. The results presented in this paper indicate that the HEAE possesses relatively strong DPPH radical and superoxide anion radical scavenging activities, while the reducing power was less pronounced. Similar to our obtained data, numerous researchers discovered a relatively high antioxidant activity in many species of mushrooms (Boonsong et al., 2016[6]; Kosanić et al., 2016[21]; Özdal et al., 2019[33]). Although there are no studies about the antioxidative activity of* H. eburneus, *our results certainly contribute to the good prospects of using this mushroom as a natural antioxidant agent.

The significance of mushrooms in cancer treatments has been constantly confirmed in recent years indicating that mushrooms can be recognized as important anticancer agents. Thus, our study represents HEAE as a possible source of anticancer compounds.

The tested extract showed cell-selective effects between colorectal and breast carcinoma, implying that HCT-116 cell line is more responsive to treatment and this sensitivity was already reported in earlier studies (Kang et al., 2016[16]; Šeklić et al., 2016[39]). According to the criteria of the American National Cancer Institute (Itharat et al., 2004[14]), significant cytotoxic activity for crude extracts is considered to be lower than 30 μg/mL, therefore HEAE with IC_50_^72h^=178.54 μg/mL expressed no significant cytotoxicity on tested cancer cell lines. Since there is no data for HEAE*, *we compared our data to cytotoxic effects of extracts of freshly collected, wild, edible mushrooms (*Lactarius deliciosus, Macrolepiota procera, Agaricus campestris, Boletus edulis*) on colon carcinoma cell lines LS174 (Kosanić et al., 2016[21], 2017[20]). It is obvious that in our study HEAE induced weaker cytotoxicity on colorectal cancer cell lines. However, when compared to IC_50_ values of extracts of collected *Fomitopsis pinicola *(Wang et al., 2014[45]) and five other, commercially purchased, medicinal mushrooms (*Phellinus linteus, Cordyceps sinensis*, *Lentinus edodes, Coprinus comatus *and* Ganoderma lucidum) *tested on HCT-116 cells, as previously published (Šeklić et al., 2016[39]), we noted that HEAE exerted better cytotoxicity.

According to the presented data, cytotoxic effects of HEAE resulted from induced apoptosis/necrosis in cancer cell lines. The results obtained by AO/EB staining which detected viable and early apoptotic cells closely relate to viable cells detected by MTT test (early apoptotic cells are alive since they metabolize tetrazolium salt used in MTT assay). However, changes in molecules that process these salts lead to late apoptosis and necrosis (Bačkorová et al., 2012[2]), while cytotoxic effect was reflected as the percentage of late apoptotic and necrotic cells.

Few repotrs have analyzed secondary metabolites found in fruiting bodies of the genus *Hygrophorus* including common fungal sterol ergosterol and its derivatives, muscaflavine, hygrophoric acid, indole, 3-chloroindole, fungicidal cyclopentenone derivatives, as well as eight bioactive fatty acids isolated from *H. eburneus* only. Wang et al. (2014[45]) already showed that ergosterol showed proapoptotic effect on human colorectal cancer cells, therefore this same active compound could probably exert the same activity in our study provoking apoptosis as the preferred type of cell death, thus showing a good anticancer effect on colorectal carcinoma HCT-116 cells.

Several studies in literature have already been focused on the effects of whole mushroom extracts on migration of cancer cells (Kim et al., 2013[18]; Wang et al., 2014[45]; Šeklić et al., 2016[39]). In this regard, our results represent the first study of *H. eburneus *antimigratory potential on cancer cell lines. Previous investigations confirmed that active substances, such as flavonoids and polyphenols, as well as ergosterol, present in mushroom extracts, could suppress the migration of different cancer cells, comprising better effects on colorectal cancer cells (Wang et al., 2014[45]; Šeklić et al., 2016[39]), being also the result of our study. Flavonoid and polyphenol contents are able to cause excessive amounts of ROS, which can affect cell motility (Šeklić et al., 2016[39]) whereas on the other hand, ergosterol showed the inhibition of colorectal carcinoma cell migration. HEAE, as well as previously tested mushroom extracts, is most likely capable of modulating signal pathways in HCT-116 cells, such as Wnt signaling components, responsible for regulating cell proliferation and migration, as an earlier study reported (Šeklić et al., 2016[39]). Our results showed that, based on presented data, HEAE induced good anticancer effect on colorectal carcinoma HCT-116 cells. Namely, it is well known that variations in the sensitivity of different cancer cell lines, being characterized by various gene expression profiles and metabolic composition, could be a factor that influences the way cells respond to natural compounds with inhibitory potential (De Los Reyes et al., 2018[7]).

Triple negative breast cancer cell lines, derived from metastatic site MDA-MB-231 cells, express low sensitivity and multidrug resistance (Theodossiou et al., 2019[42]), which can be used as an explanation for the weak cytotoxicity of HEAE detected in this cell line. Apoptosis noted in our treatments showed that tested extract had certain effects on cell death, suggesting that active substances present in HEAE obviously affected MDA-MB-231 cells. However, multidrug resistance of this cell line did not permit HEAE to induce notable cytotoxicity, therefore, the combination of HEAE with standard chemotherapeutic agents, such as bevacizumab, tamoxifen or paclitaxel (Tong et al., 2018[43]) could be a promising therapy for resistant types of breast cancer. Although these cells are characterized by their high invasiveness and enhanced basal migration (Kazan et al., 2019[17]; Theodossiou et al., 2019[42]), we detected weak antimigratory effects of HEAE in concentration of 10 µg/mL after 12 h. 

In conclusion, the present study can be stated to have special importance considering the fact that there has been no information regarding bioactive properties of *H. eburneus*, the edible mushroom. According to the obtained results, *H. eburneus* might be used as a novel natural product to promote human health through its neuroprotective, antimicrobial, antioxidant, and anticancer activities. Further studies should be aimed at isolation, characterization and testing of individual bioactive compounds from the selected mushroom. Also, more detailed *in vitro* as well as *in vivo* studies and investigation of these mechanisms are needed. 

## Acknowledgements

This study was supported by the Ministry of Education, Science and Technological Development of the Republic of Serbia (project nos. 173032 and III41010).

## Conflict of interest

The authors declare that they have no conflict of interest.

## Supplementary Material

Supplementary data

## Figures and Tables

**Table 1:**
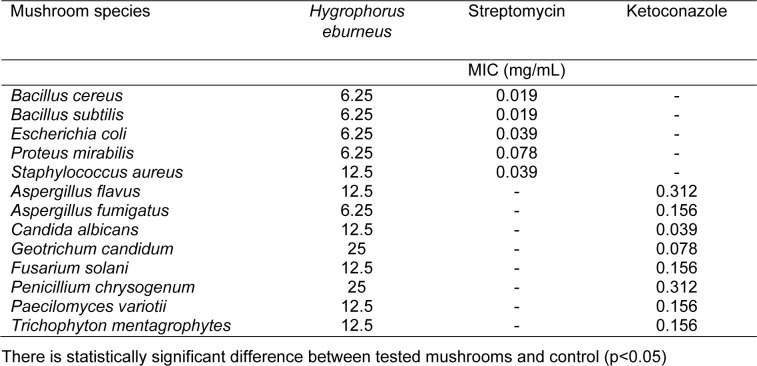
Minimum inhibitory concentration (MIC) of HEAE

**Table 2:**
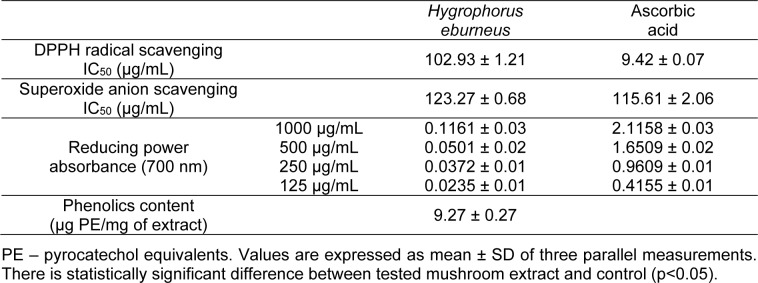
DPPH radical scavenging activity, superoxide anion scavenging activity, reducing power and phenolics content of HEAE

**Table 3:**
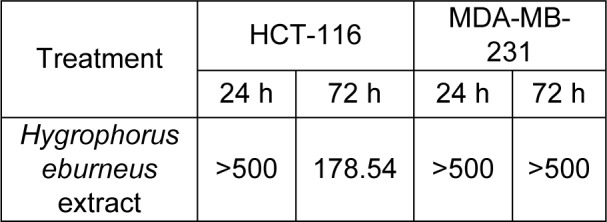
Cytotoxic effects - IC_50_ values (μg/mL) of HEAE on HCT-116 and MDA-МB-231 cell lines

**Table 4:**
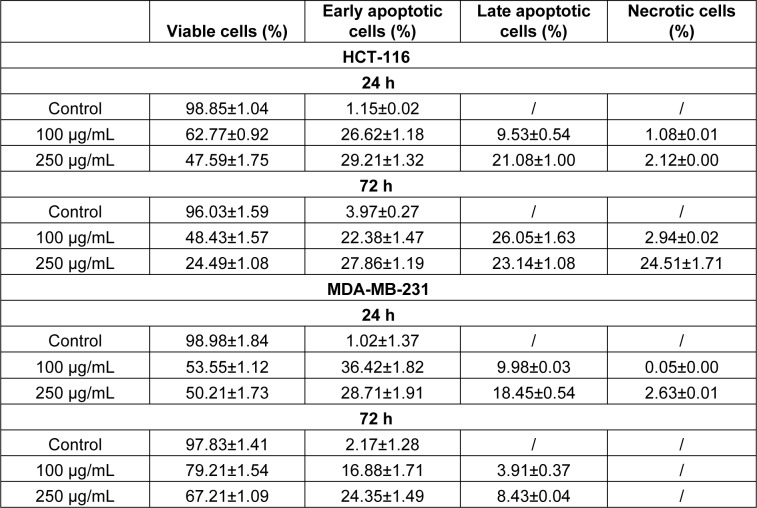
Percentages of viable, apoptotic and necrotic HCT-116 and MDA-MB-231 cells measured with AO/EB fluorescence staining, after treatment with HEAE. Results are presented as means ± SE of two independent experiments.
