# Supplementary material for: Hygrophorus eburneus, edible mushroom, a promising natural bioactive agent
Source: EXCLI J. 2020 Mar 31;19:442–57. doi: 10.17179/excli2019-2056 (PMC7214781; doi:10.17179/excli2019-2056)
Supplement: Supplementary data [file EXCLI-19-442-s-001.pdf]

EXCLI Journal 2020;19:442-457 – ISSN 1611-2156
Received: December 11, 2019, accepted: March 23, 2020, published: March 31, 2020

Supplementary data to:

HYGROPHORUS EBURNEUS, EDIBLE MUSHROOM, A PROMISING NATURAL BIOACTIVE AGENT

Marijana M. Kosanić*, Dragana S. Šeklić, Milena M. Jovanović, Nevena N. Petrović, Snežana D. Marković

Faculty of Science, Department of Biology and Ecology, University of Kragujevac, Radoja Domanovića 12, 34000 Kragujevac, Serbia

* **Corresponding author:** Marijana M. Kosanić, Faculty of Science, Department of Biology and Ecology, University of Kragujevac, Radoja Domanovića 12, 34000 Kragujevac, Serbia, Tel: +38134336223; Fax: +38134335040; E-mail: marijanakosanic@yahoo.com

http://dx.doi.org/10.17179/excli2019-2056

This is an Open Access article distributed under the terms of the Creative Commons Attribution License (http://creativecommons.org/licenses/by/4.0/).

Supplementary Table 1: Observed absorbances of effect of HEAE (in concentration range from 125 to 1000 µg/mL) in acetylcholinesterase inhibition (Raw data concerning **Figure 1**)

	Absorbance (412 nm)			
	Concentration (µg/mL)	n1	n2	n3
Control	*0*	2.688	2.687	2.691
Hygrophorus eburneus	*1000*	1.521	1.443	1.358
	500	1.774	1.703	1.841
	250	2.054	1.999	2.105
	125	2.334	2.367	2.301
Galanthamine	*1000*	0.143	0.107	0.376
	500	0.247	0.311	0.483
	250	0.654	0.606	0.811
	125	0.948	0.778	0.846

EXCLI Journal 2020;19:442-457 – ISSN 1611-2156
Received: December 11, 2019, accepted: March 23, 2020, published: March 31, 2020

Supplementary Table 2: Observed absorbances of effects of HEAE (in concentration range from 1 to 500 µg/mL) on viability of HCT-116 and MDA-MB-231 cells (Raw data concerning **Figure 2**)

Time of treatment	Concentration (µg/mL)													
	control *0*	*1*	*10*	*50*	*100*	*250*	*500*	control *0*	*1*	*10*	*50*	*100*	*250*	*500*
**24 h**	HCT-116							MDA-MB-231						
	0.468	0.568	0.617	0.576	0.441	0.456	0.234	1.462	1.254	1.221	1.314	1.21	1.244	1.209
	0.419	0.502	0.666	0.355	0.449	0.316	0.306	1.407	1.068	1.017	1.116	1.062	1.293	1.283
	0.381	0.499	0.591	0.618	0.5	0.398	0.252	1.128	1.315	1.121	1.086	1.267	1.238	1.222
	0.387	0.507	0.6	0.433	0.319	0.362	0.363	1.389	1.12	1.222	1.163	1.204	1.246	1.218
	0.553	0.583	0.488	0.555	0.377	0.282	0.312	1.541	1.192	1.323	1.353	1.311	1.3	1.078
	0.692	0.545	0.386	0.474	0.446	0.486	0.302	1.589	1.651	1.487	1.265	1.317	1.102	1.249
	0.443	0.553	0.397	0.49	0.421	0.361	0.289	1.622	1.282	1.399	1.20	1.224	1.203	1.234
	0.597	0.539	0.509	0.513	0.446	0.359	0.275	1.887	1.126	1.103	1.17	1.12	1.14	1.19
	0.642	0.557	0.541	0.503	0.399	0.404	0.321	1.418	1.391	1.2	1.19	1.20	1.18	1.12
**72 h**	HCT-116							MDA-MB-231						
	2.115	1.232	1.8	1.994	2.223	0.977	0.246	2.03	2.041	2.017	1.892	2.265	2.049	1.259
	2.028	1.596	1.771	1.64	1.292	0.784	0.246	1.944	1.952	1.962	1.789	1.795	1.916	1.661
	2.191	1.865	1.732	1.974	1.515	0.868	0.291	2.219	1.738	2.101	1.898	1.609	1.903	1.409
	2.201	1.967	1.945	1.787	1.341	0.822	0.276	1.669	2.057	2.251	1.977	1.957	2.01	1.557
	2.216	2.216	1.997	1.912	1.119	0.52	0.239	2.158	1.933	1.931	2.116	2.13	1.865	1.807
	1.608	2.028	1.802	1.599	1.618	0.777	0.227	1.93	1.909	2.106	2.157	2.029	1.907	1.603
	2.35	2.075	1.719	1.823	1.495	0.795	0.202	1.826	1.989	1.89	1.993	1.875	1.865	1.573
	1.526	1.914	1.813	1.787	1.52	0.815	0.292	2.027	2.281	1.921	1.921	1.89	1.922	1.506
	2.1	1.862	1.822	1.899	1.539	0.765	0.269	1.807	2.251	1.818	2.031	2.011	2.109	1.569

EXCLI Journal 2020;19:442-457 – ISSN 1611-2156
Received: December 11, 2019, accepted: March 23, 2020, published: March 31, 2020

Supplementary Table 3: The effect of HEAE on migratory potential of HCT-116 cells and MDA-MB-231 presented as measurements of wound space by ImageJ software (Raw data concerning **Figures 3** and **5**)

Time of treatment	Concentration (µg/mL)					
	control *0*	*10*	*100*	control *0*	*10*	*100*
**0 h**	HCT-116			MDA-MB-231		
	1.237	1.363	1.285	1.28	1.155	1.356
	1.283	1.453	1.295	1.253	1.155	1.201
	1.064	1.245	1.295	1.335	1.146	1.301
	1.41	1.326	1.358	1.317	1.128	1.228
	1.283	1.099	1.213	1.371	1.137	1.365
	1.228	1.317	1.213	1.317	1.283	1.237
**12 h**	HCT-116			MDA-MB-231		
	0.628	0.581	0.893	0.036	0.055	0.055
	0.582	0.6	1.003	0.055	0.055	0.064
	0.664	0.536	0.784	0.045	0.055	0.055
	0.619	0.636	0.866	0.054	0.073	0.045
	0.573	0.627	0.839	0.036	0.046	0.055
	0.682	0.636	0.811	0.036	0.046	0.038
**24 h**	HCT-116			MDA-MB-231		
	0.246	0.409	0.647	0.029	0.036	0.036
	0.309	0.391	0.702	0.009	0.027	0.029
	0.309	0.409	0.693	0.029	0.055	0.027
	0.209	0.391	0.611	0.027	0.027	0.029
	0.246	0.3	0.638	0.018	0.027	0.036
	0.3	0.372	0.729	0.027	0.036	0.027

EXCLI Journal 2020;19:442-457 – ISSN 1611-2156
Received: December 11, 2019, accepted: March 23, 2020, published: March 31, 2020

Supplementary Table 4: Number of HCT-116 and MDA-MB-231 cells observed after HEAE exposure (in concentrations 100 and 250 µg/mL) (Raw data concerning **Figure 4**)

	Viable cells	Early apoptotic cells	Late apoptotic cells	Necrotic cells	Σ
		HCT-116			
		**24 h**			
**0 µg/mL**	445	5	0	0	450
	305	4	0	0	309
**100 µg/mL**	223	98	34	4	356
	193	82	29	3	307
**250 µg/mL**	261	160	116	12	549
	203	125	90	9	427
		**72 h**			
**0 µg/mL**	534	22	0	0	556
	422	17	0	0	439
**100 µg/mL**	261	120	140	16	537
	243	112	132	15	502
**250 µg/mL**	97	111	92	98	398
	98	112	93	98	401
		MDA-MB-231			
		**24 h**			
**0 µg/mL**	421	4	0	0	425
	318	3	0	0	321
**100 µg/mL**	190	129	35	1	355
	208	142	39	0	389
**250 µg/mL**	154	88	56	8	306
	159	91	58	8	317
		**72 h**			
**0 µg/mL**	499	11	0	0	510
	314	7	0	0	321
**100 µg/mL**	325	69	16	0	410
	294	63	14	0	371
**250 µg/mL**	232	84	29	0	345
	282	102	35	0	419

EXCLI Journal 2020;19:XX-XX – ISSN 1611-2156
Received: XX, 2019, accepted: XX, 2019, published: XX, 2020

Supplementary Table 5: Observed absorbances of effects of HEAE in DPPH radical scavenging activity, superoxide anion scavenging activity, reducing power, and phenolics content (Raw data concerning **Table 2**)

	**DPPH radical scavenging activity (Absorbance 517 nm)**												
	Control	*Hygrophorus eburneus*						Ascorbic acid					
Concentration (µg/mL)	*0*	*1000*	*500*	*250*	*125*	*62.5*	*31.2*	*100*	*50*	*25*	*12.5*	*6.25*	*3.12*
n1	1.0270	0.0256	0.0307	0.3203	0.4590	0.5782	0.7415	0.0239	0.0454	0.1471	0.3904	0.6497	0.8164
n2	1.0936	0.0297	0.0395	0.2996	0.4983	0.5995	0.8298	0.0254	0.0609	0.1572	0.4199	0.6989	0.8727
n3	1.0553	0.0291	0.0361	0.3009	0.4889	0.5895	0.7695	0.0251	0.0845	0.1727	0.3946	0.6793	0.8184
	**Superoxide anion scavenging activity (Absorbance 560 nm)**												
	Control	*Hygrophorus eburneus*						Ascorbic acid					
Concentration (µg/mL)	*0*	*1000*	*500*	*250*	*125*	*62.5*	*31.2*	*1000*	*500*	*250*	*125*	*62.5*	*31.2*
n1	0.7553	0.0411	0.1232	0.1931	0.2991	0.4242	0.6095	0.0401	0.1224	0.1928	0.2992	0.4144	0.6019
n2	0.7596	0.0391	0.1259	0.1983	0.3005	0.4251	0.6102	0.0388	0.1251	0.1981	0.2095	0.4219	0.6092
n3	0.7591	0.0389	0.1257	0.1985	0.3011	0.4241	0.6117	0.0391	0.1260	0.1908	0.2995	0.4203	0.6120

	**Reducing power (Absorbance 700 nm)**							
	Hygrophorus eburneus				Ascorbic acid			
Concentration (µg/mL)	*1000*	*500*	*250*	*125*	*1000*	*500*	*250*	*125*
n1	0.0971	0.0699	0.0381	0.0299	2.1475	1.6509	0.9611	0.4174
n2	0.1511	0.0401	0.0312	0.0206	2.1001	1.6719	0.9696	0.4199
n3	0.1001	0.0404	0.0433	0.0201	2.0998	1.6301	0.9521	0.4091
	**Phenolics content (Absorbance 760 nm)**							
n1	0.0361							
n2	0.0349							
n3	0.0381							
